# Differences in white blood cell proportions between schizophrenia cases and controls are influenced by medication and variations in time of day

**DOI:** 10.1038/s41398-023-02507-1

**Published:** 2023-06-17

**Authors:** Jonelle D. Villar, Anne-Kristin Stavrum, Leticia M. Spindola, Anja Torsvik, Thomas Bjella, Niels Eiel Steen, Srdjan Djurovic, Ole A. Andreassen, Vidar M. Steen, Stephanie Le Hellard

**Affiliations:** 1 NORMENT, Department of Clinical Science, University of Bergen, Bergen, Norway; 2 Dr. Einar Martens Research Group for Biological Psychiatry, Department of Medical Genetics, Haukeland University Hospital, Bergen, Norway; 3 NORMENT, Division of Mental Health and Addiction, Oslo University Hospital and Institute of Clinical Medicine, University of Oslo, Oslo, Norway

**Keywords:** Genomics, Physiology

## Abstract

Cases with schizophrenia (SCZ) and healthy controls show differences in white blood cell (WBC) counts and blood inflammation markers. Here, we investigate whether time of blood draw and treatment with psychiatric medications are related to differences in estimated WBC proportions between SCZ cases and controls. DNA methylation data from whole blood was used to estimate proportions of six subtypes of WBCs in SCZ patients (*n* = 333) and healthy controls (*n* = 396). We tested the association of case-control status with estimated cell-type proportions and the neutrophil-to-lymphocyte ratio (NLR) in 4 models: with/without adjusting for time of blood draw, and then compared results from blood samples drawn during a 12-h (07:00–19:00) or 7-h (07:00-14:00) period. We also investigated WBC proportions in a subgroup of medication-free patients (*n* = 51). Neutrophil proportions were significantly higher in SCZ cases (mean=54.1%) vs. controls (mean=51.1%; *p* = <0.001), and CD8^+^T lymphocyte proportions were lower in SCZ cases (mean=12.1%) vs. controls (mean=13.2%; *p* = 0.001). The effect sizes in the 12-h sample (07:00–19:00) showed a significant difference between SCZ vs. controls for neutrophils, CD4^+^T, CD8^+^T, and B-cells, which remained significant after adjusting for time of blood draw. In the samples matched for time of blood draw during 07.00–14.00, we also observed an association with neutrophils, CD4^+^T, CD8^+^T, and B-cells that was unaffected by further adjustment for time of blood draw. In the medication-free patients, we observed differences that remained significant in neutrophils (*p* = 0.01) and CD4^+^T (*p* = 0.01) after adjusting for time of day. The association of SCZ with NLR was significant in all models (range: *p* < 0.001 to *p* = 0.03) in both medicated and unmedicated patients. In conclusion, controlling for pharmacological treatment and circadian cycling of WBC is necessary for unbiased estimates in case-control studies. Nevertheless, the association of WBC with SCZ remains, even after adjusting for the time of day.

## Introduction

Schizophrenia (SCZ) is a debilitating psychiatric disorder with predominantly unknown disease mechanisms. Several lines of evidence implicate the immune system, and increases in total white blood cell (WBC) proportions are seen in individuals with SCZ. Hannon [1] et al. report distinct cellular compositional changes and identify the association of increased myeloid cells (granulocytes and monocytes) and decreased lymphoid cells (CD4^+^T, CD8^+^T, NK cells) with a lengthy course of illness. In first-episode psychosis (FEP), however, mean differences in these cells were null or minimal compared to established SCZ cases [1]. Reduced WBC counts are reported after the initiation of antipsychotic (AP) drug treatment. Nevertheless, metabolic disturbances accompanying these drugs may account for increased WBC counts over time [2]. Additional factors may contribute to alterations in WBC proportions, including low-grade persistent inflammatory abnormalities, poor physical health, diet, polypharmacy, and the psychosocial stress experienced by living with the disorder [3].

Circadian rhythms exert an oscillating influence on WBC activity [4, 5], which are often disrupted in SCZ [6], as evidenced by sleep disorders prior to [7, 8] and following remission [9]. The question is raised, therefore, whether circadian fluctuations in cell proportions affect blood-based results of SCZ when study designs have not adjusted for the influence of time of day. One such omic approach is an epigenome-wide association study (EWAS) that seeks to identify differential DNA methylation (DNAm) between cases and controls at cytosine nucleotides. For instance, Oh [10] et al. show that previously reported differences in DNAm identified in large EWASs of SCZ versus controls partially coincide with DNAm differences associated with time of day identified in the general population. The results from their model strongly suggest that differences in blood-draw time between cases and controls can prompt a false association of blood cell counts with the disorder, and inadvertently, the association between DNAm or cell type could be attributed to actual differences between cases and controls [10].

Studies included in meta-analyses often have different study designs with potentially distinct routines for sample collection. For instance, some studies include control samples from blood banks, commonly with a broader time range of blood collection, while cases are usually recruited in a clinical setting with blood collection taken in the morning. In the present study, we use DNA methylation data from whole blood to estimate WBC proportions in SCZ cases and healthy controls and examine the influence of time of day when blood was drawn. We further our investigation of the time of day effect by evaluating the neutrophil-to-lymphocyte (NLR) ratio, previously shown to detect inflammatory activation related to SCZ, related psychoses [11–13], and psychotropic treatment response [13, 14]. We compare these results with those of a sub-group of medication-free SCZ cases to estimate the impact of polypharmacy on WBC.

## Methods

Patients with schizophrenia and healthy controls were recruited through the Thematically Organised Psychosis (TOP) study cohort at the Norwegian Centre for Mental Disorders Research (NORMENT, Oslo, Norway; (https://www.med.uio.no/norment/english). Diagnoses were obtained by trained clinical psychologists or physicians using the Structured Clinical Interview for DSM-IV Axis I Disorders (SCID-I) [15] with an 82% diagnostic reliability for DSM-IV diagnostic categories [16]. History of psychosis was retrieved from SCID and clinical interviews. DSM_IV codes for SCZ (295.10–295.90) represented 80% of cases selected in this study, followed by Other Psychotic Disorders (297.10 and 298.90). Pharmacological treatment was obtained from medical records and patient interviews and confirmed with serum level assessments to clarify non-compliance [17]. Polypharmacy was defined as the simultaneous use of 2 or more antipsychotic medications and may include mood stabilizers, antidepressants, anxiolytics, hypnotics [18], and other somatic treatments [19]. Healthy controls were recruited by application from the Statistics Norway (SSB) Register and included individuals living in Oslo and nearby areas.

**Fig. 1:**
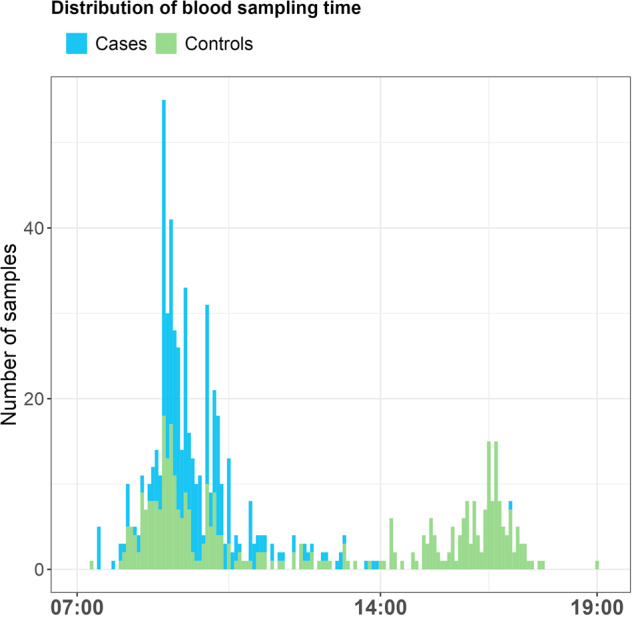
Distribution of time of blood draw for cases and controls during a typical working day (07:00–19:00). SCZ cases (seen in blue) have recorded blood draws between 07:00 and 14:00 while controls (seen in green) have recorded blood draws between 07:00 and 19:00. This distribution suggests the potential bias that may occur when time of blood draw is not matched between cases and control.

Here we included patient blood samples collected between 07:00 and 14:00 (7-h sample), which is typical for clinical samples, and samples collected between 07:00 and 19:00 (12-h sample) for controls, reflecting a typical time range due to work and studies. See Fig. 1 for the distribution of time of blood draw. All blood samples were stored in the TOP biobank. For the analysis, samples were selected from participants aged 18–48 years, and cases and controls were matched for age (median age = 31.0 years) and sex (58% male). The total sample size (*n* = 729) included a subset of medication-free cases (*n* = 51, median age = 28.0 years, 67% male). Further demographics are included in Supplementary Tables 1–4. Only samples with recorded time of blood draw were included.

### DNA methylation quantification

DNA from peripheral blood samples was bisulfite-converted, which enables the identification of methylated versus unmethylated cytosines. Methylated cytosines are unchanged by the sodium bisulfite, while unmethylated cytosines are converted to uracils and amplified by PCR as thymines. The DNA was then assayed on the Infinium® MethylationEPIC BeadChip (Illumina Inc., CA, USA), which interrogates over 850 K CpGs across the genome and quantifies the methylation levels at those sites. The typing was performed at the Institute of Human Genetics, University Hospital of Bonn, Germany.

### Pre-processing and quality control

Quality control (QC) and preprocessing of the DNAm data was performed using R Statistical Software, version 4.0.0, R Core Team (2020) [20] and Bioconductor (version 3.1.1) packages: {minfi} [21], {watermelon} [22], {sva} [23], and {ChAMP} [24].

The DNAm data were generated in three batches of 1000, 283, and 1082 samples as the data became available. QC and preprocessing were performed for each batch separately. The filtering of probes was based on the following criteria: (1) removed probes with a detection *P*-value > 0.01 in more than 1% of samples; (2) removed probes with a bead count <3 in more than 5% of the samples; (3) removed probes known for cross-hybridization or for containing a single nucleotide polymorphism (SNP) mapped close to the 3′-end of the probe (the target CpG), as reported in Zhou [25] et al.; and (4) removed probes mapped on the sex chromosomes. Similarly, for samples, the filtering was based on the following criteria: (5) excluded samples having more than 1% of probes with a detection *P*-value > 0.01; (6) excluded samples that showed a mismatch between (6a) reported and predicted sex based on DNAm data; (6b) the 59 SNP genotypes obtained from the EPIC array and the genotypes obtained from SNP data; and (6c) reported and predicted ethnicity. Ethnicity was predicted from genetic data using a Random Forest classifier, and the model was trained using populations from the 1000 Genomes Project [26].

To reduce non-biological variation, data were normalized using functional normalization [27]. Twenty principal components (PCs) were used to normalize the first and second batches, and 25 for the third batch. {Combat} [28] was used to remove the batch effects from technical sources, including sentrix ID, sentrix position, and scanner ID. The total number of samples removed due to failed QC checks was *n* = 63, and the combined number of samples removed due to failed genotype check, or which revealed a mismatch between predicted and reported sex or ethnicity was *n* = 76.

After QC and preprocessing, the data from the three typing batches were merged. {ComBat} [28] was used to remove typing batch effects. Visualization of technical replicates between the different typing batches on a PCA plot confirmed that the technical replicates appeared close. This validated the QC and preprocessing procedures.

Cell-type proportions in 6 constituent cell types (monocytes, neutrophils, natural killer cells (NK), CD4^+^T, CD8^+^T, and B-cells) were estimated from DNAm data using the Houseman [29] algorithm as implemented in the *estimateCellCounts2* function from the R package {FlowSorted.Blood.Epic} [30], using default settings. The quality of the estimated cell-type proportions was then evaluated by calculating the Deconvolution Specific Root Mean Squared Error (DSRMSE) as described by Vellame [31] et al. We calculated the error per sample (1818 available samples with an error range 0.0334–0.0908 and removed 164 samples with a DSRMSE > 0.06.

Finally, the subset of samples from cases and controls was extracted and used for the analyses. The final data set had 729 samples and 760,668 probes. A singular value decomposition (SVD) analysis implemented from {ChAMP} [24] was performed on our selected samples dataset to identify variation from known technical batch effects that should be adjusted for in the models in the subsequent statistical analysis.

### Statistical analysis

All analyses were performed using R Statistical Software, version 4.0.3, R Core Team (2020) [20].

We divided our analyses into two samples. The 12-h sample included all individuals with recorded blood draws between 07:00 and 19:00 (*n* = 729): SCZ cases (*n* = 333, male 57%) vs. controls (*n* = 396, male 58%), while the 7-h sample included individuals with recorded blood draws between 07:00 and 14:00 (*n* = 564): SCZ cases (*n* = 332, male 57%) vs. controls (*n* = 232, male 60%). A subset of medication-free SCZ cases was investigated by comparing a 12-h subset: SCZ cases (*n* = 51, male 67%) vs. controls (*n* = 315, male 62%) to the 7-h subset: SCZ cases (*n* = 49, male 65%) vs. controls (*n* = 184, male 62%).

We performed a residual analysis by linear regression of each cell type against the largest technical batch effect (AMP plate), e.g., *lm(Mono ~ AMP_plate)*. Multivariable linear regression was then performed with the residuals from each cell type incorporated as the dependent variable and included in the model the biological and environmental covariates (age, sex, smoking score) and phase of methylation typing (Methbatch), which was the second largest technical batch effect. As smoking status was not recorded for all samples, a smoking score was calculated from methylation data using the method described by Hannon [32] et al. *P*-values were corrected for multiple testing using the Benjamini-Hochberg procedure.

#### Model 1: unadjusted for time of day

$$\begin{array}{l}Cell - type\;proportions\;Case\_Control + Age + Sex \\ \qquad+ \,Smoking\_score + Methbatch + \varepsilon\end{array}$$

#### Model 2: adjusted for time of day

The time variable for blood collection was converted to represent the difference in hours from baseline (07:00) and incorporated into the fully adjusted model, e.g.,

$$\begin{array}{l}Cell - type\;proportions\;Case\_Control\\ \qquad+ \,Age + Sex + Smoking\_score + Methbatch\\ \qquad+ \,Hours\_from\_baseline + \varepsilon\end{array}$$

#### Neutrophil-to-lymphocyte ratio

The NLR was calculated by dividing the estimated proportions of neutrophils by the estimated proportions of NK, CD4^+^T, CD8^+^T, and B cells. The distribution of NLR values was inspected visually using a histogram plot for potential outliers, as shown in Supplementary Fig. 1. The association of SCZ cases vs. controls with NLR was estimated using the unadjusted and adjusted models and included biological covariates and the batch effect for AMP plate.

## Results

An evaluation of the distribution of blood-draw times of the samples selected from our larger cohort shows a bias in blood-draw sampling times between cases and controls, see Fig. 1. When the time frame is shortened from 12 to 7 h, the blood-draw sampling times follow similar distributions, with cases and controls more evenly matched (see Supplementary Figures 2 and 3). The 12-h and 7-h samples represent likely common scenarios when the time of blood draw is considered or not in experimental designs. Therefore, we compared the 12-h and 7-h samples in our analyses to investigate the latent contribution of the time of blood draw on our association study of SCZ on WBC proportions.

### Effect of time of day on the differences in WBC proportions between cases and controls

In the first analysis, we evaluated the effect of time of day on the association between WBC proportions and 12-h cases vs. controls by not adjusting or adjusting for time of blood draw.

#### WBC

*For the 12-h sample (07:00–19:00)*, *β*-estimates and *p*-values from multivariable linear regression show differences between SCZ cases and controls for neutrophils, CD4^+^T, CD8^+^T, and B-cell proportions after adjusting for age, sex, smoking score, and technical batch. The strength of association was diminished after adjusting for the time of blood draw (see Table 1 and Supplementary Table 5 for standard errors). *For the 7-h sample (07:00–14:00)*, differences were seen in the same cell types reported in the 12-h sample and remained unchanged after adjusting for the time of blood draw (see Table 1 and Supplementary Table 6 for standard errors).

**Table 1:** *β*-estimates and *p*-values for cell types and mean NLR derived from regression models unadjusted and adjusted for time of blood draw in two time periods for SCZ vs. controls.

Covariates	Stats	Mono	Neu	NK	CD4 + T	CD8 + T	B cell	NLR
12 h - 07:00–19:00 - cases (*N* = 333) vs. controls (*N* = 396)								
Age, sex, smoking, technical	beta	0.0008	**0.03**	−0.003	**−0.01**	**−0.01**	**−0.005**	**0.17**
	pval	0.63	**<0.001**	0.09	**0.006**	**0.001**	**0.004**	**<0.001**
Age, sex, smoking, technical, blood-draw time	beta	−0.0005	**0.03**	−0.002	**−0.01**	**−0.01**	**−0.004**	**0.16**
	pval	0.78	**0.003**	0.29	**0.02**	**0.01**	**0.03**	**0.01**
7 h - 07:00–14:00 - cases (*N* = 332) vs. controls (*N* = 232)								
Age, sex, smoking, technical	beta	−0.0006	**0.03**	−0.001	**−0.01**	**−0.01**	**−0.005**	**0.14**
	pval	0.74	**0.008**	0.51	**0.02**	**0.04**	**0.02**	**0.02**
Age, sex, smoking, technical, blood-draw time	beta	−0.0006	**0.03**	−0.001	**−0.01**	**−0.01**	**−0.004**	**0.14**
	pval	0.76	**0.009**	0.52	**0.02**	**0.04**	**0.02**	**0.02**

Cell types with significant differences are in bold.

**Fig. 2:**
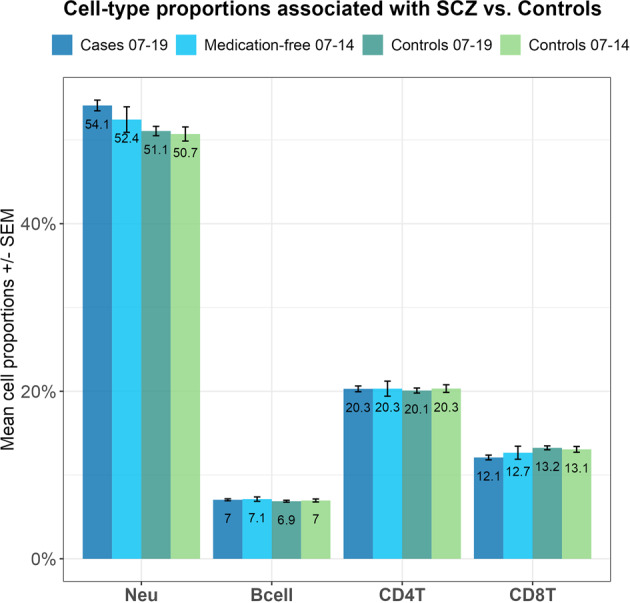
Representation of mean cell-type proportions that significantly differed in SCZ cases vs. controls. For the 12-h cases (*n* = 333) vs. controls (*n* = 396) seen in Table 1, neutrophils, CD4^+^T, CD8^+^T, and B-cells were significantly different. Here neutrophil proportions were higher for cases (mean = 54.1%, 95% CI 0.53–0.55) than for controls (mean = 51.1%, 95% CI 0.51–0.52). For 7-h medication-free cases (*n* = 49) vs. controls (*n* = 184) seen in Table 2, only neutrophils and CD4^+^T cells were significantly different. Here neutrophil proportions remained higher for cases (mean = 52.4, 95% CI 0.50–0.55) than for controls (mean = 50.7%, 95% CI 0.50–0.52).

The largest differences in mean proportions were seen in neutrophil and CD8^+^T lymphocyte proportions, respectively (see Fig. 2). Neutrophil proportions in 12-h SCZ cases (mean = 54.1%) vs. controls (mean = 51.1%), and CD8^+^T lymphocyte in 12-h SCZ cases (mean = 12.1%) vs. controls (mean = 13.2%). For the subgroup of medication-free cases, mean differences were also largest for neutrophil proportions in SCZ cases (mean = 52.4%) vs. controls (mean = 50.7%), although reduced for CD8^+^T lymphocyte proportions in SCZ cases (mean = 12.7%) vs. controls (mean = 13.1%).

### Effect of time of day on the differences in NLR between cases and controls

In the second analysis, we evaluated whether time of day modified the association between NLR and 12-h cases vs. controls by not adjusting or adjusting for time of blood draw. Two samples were removed as outliers following a visual inspection of the histogram (one case NLR = 4.37 and one control NLR = 6.25) (Supplementary Figure 1).

#### NLR

*For the 12-h sample (07:00–19:00)*, *β*-estimates and *p*-values from multivariable linear regression show a significant association for SCZ vs. controls whether we adjusted for time of blood draw or not; unadjusted NLR = 0.17, SE = 0.05, 95% CI [0.07, 0.27], *p* < 0.001), and adjusted NLR = 0.16, SE = 0.06, 95% CI [0.04, 0.27], *p* = 0.01. *For the 7-h sample (07:00-14:00)*, SCZ remained significantly associated with NLR vs. controls and did not change after adjusting for time of blood draw; unadjusted NLR = 0.14, SE = 0.06, 95% CI [0.03, 0.26], *p* = 0.02) and adjusted NLR = 0.14, SE = 0.06, 95% CI [0.02, 0.26], *p* = 0.02; see Table 1).

### Effect of time of day on WBC proportions and NLR in unmedicated cases vs. controls

In the final analysis, we evaluated the effect of time of day on the association between WBC proportions and NLR by not adjusting or adjusting for time of blood draw. Here we investigated a subset of unmedicated cases vs. controls.

#### WBC

For the 12-h sample (07:00–19:00), *β*-estimates and *p*-values from multivariable linear regression revealed that the association between medication-free cases and neutrophils and CD4^+^T lymphocytes remained significant, yet slightly moderated after adjusting for blood-draw time. There was no significant observable association for monocytes or other lymphocytes (see Table 2 and Supplementary Table 7 for standard errors).

**Table 2:** *β*-estimates and *p*-values for cell types and mean NLR derived from regression models unadjusted and adjusted for time of blood draw in two time periods for medication-free cases vs. controls.

Covariates	Stats	Mono	Neu	NK	CD4 + T	CD8 + T	B cell	NLR
12 h - 07:00–19:00 - medication-free cases (*N* = 51) vs. controls (*N* = 315)								
Age, sex, smoking, technical	beta	−0.009	**0.08**	−0.0009	**−0.05**	−0.02	−0.007	**0.42**
	pval	0.13	**0.007**	0.90	**0.001**	0.24	0.25	**0.01**
Age, sex, smoking, technical, blood-draw time	beta	−0.01	**0.08**	−0.002	**−0.05**	−0.01	−0.006	**0.38**
	pval	0.06	**0.01**	0.77	**0.002**	0.34	0.31	**0.03**
7 h - 07:00–14:00 - medication-free cases (*N* = 49) vs. controls (*N* = 184)								
Age, sex, smoking, technical	beta	−0.009	**0.08**	−0.0003	**−0.05**	−0.02	−0.007	**0.49**
	pval	0.17	**0.01**	0.97	**0.01**	0.12	0.30	**0.01**
Age, sex, smoking, technical, blood-draw time	beta	−0.009	**0.08**	−0.0008	**−0.05**	−0.02	−0.007	**0.47**
	pval	0.16	**0.01**	0.92	**0.01**	0.13	0.32	**0.02**

Cell types with significant differences are in bold.

#### NLR

The ratio was significant for unmedicated SCZ cases vs. controls whether we adjusted for time of blood draw or not; unadjusted NLR = 0.42 SE = 0.17, 95% CI [0.08, 0.75], *p* = 0.01) and adjusted NLR = 0.38, SE = 0.17, 95% CI [0.04, 0.71], *p* = 0.03).

#### WBC

For the 7-h sample (07:00–14:00), *β*-estimates and *p*-values from multivariable linear regression revealed that the association between medication-free cases and neutrophils and CD4^+^T lymphocytes was significant and unchanged after adjusting for blood-draw time. There was no significant observable effect for monocytes or other lymphocytes (see Table 2 and Supplementary Table 8 for standard errors).

#### NLR

*β*-estimates were slightly larger than in the 12-h sample, and the ratio remained significant for unmedicated SCZ cases vs. controls whether we adjusted for time of blood draw or not; unadjusted NLR = 0.49, SE = 0.19, 95% CI [0.11, 0.88], *p* = 0.01) and adjusted NLR = 0.47, (SE = 0.19, 95% CI [0.09, 0.84], *p* = 0.02; see Table 2).

## Discussion

In our study, we identify altered WBC proportions in SCZ cases vs. controls, and these differences were also observed in unmedicated patients. We consider the influence of circadian rhythms on WBC proportions and identify the effect of time of day on the association with SCZ cases vs. controls in samples unmatched for time of blood draw. We also identify a persistent, significant association with SCZ cases vs. controls on the NLR whether or not we adjust for time of blood draw.

The analysis of all samples revealed that four of the six estimated cell-type proportions (neutrophils, CD4^+^T, CD8^+^T, and B-cells) were significantly different in SCZ vs. healthy controls. These associations remained significant after adjusting for time of blood draw, although the effect sizes were diminished (Table 1). For these four cell-types, our findings replicate the direction of effects in cell-type proportions seen in an extensive study of cellular alterations in SCZ and FEP. Here both Hannon et al. [1] and our study identified increased proportions of neutrophils and decreased proportions of CD4^+^T, CD8^+^T, and B-cells in SCZ vs. controls. Although DNA methylation data were used to estimate these cell-type proportions, previous empirical cell studies confirm increased neutrophil counts in SCZ [12, 33].

Irrespective of poor physical health, age, sex, BMI, or smoking [34], WBC counts are significantly higher in individuals with SCZ than in healthy controls. Antipsychotic medication, in combination with other psychiatric drugs, including antidepressants and mood stabilizers, increases the risk of alterations in leukocyte proportions, particularly in neutrophils [35]. We investigated, therefore, our subset of medication-free cases to see if we could identify differences in WBC proportions compared to medicated cases. Only neutrophils and CD4^+^T cells were significantly associated with medication-free cases, with larger effect sizes seen for neutrophils than for CD4^+^T (Table 2). While circadian variation is biologically significant in both cell types [5], one must also consider that an increase in one cell-type proportion will alter the proportion of one or more cell types (Fig. 2) without actually altering the abundance of that cell type [1]. Nevertheless, effect sizes for neutrophils in the medication-free cases were larger than those seen in medicated cases. These results suggest that polypharmacy has a more significant effect on neutrophil proportions than time of blood draw. While it is interesting that the effect sizes of neutrophils and CD4 + T are larger in the unmedicated samples than in the medicated samples, we are hesitant to speculate on a biological interpretation. Our sample size is small (*n* = 51), and these findings call for replication with a larger sample size.

Further studies of WBC dysregulation in SCZ indicate the advantage of using the NLR ratio to assess systemic inflammation in psychosis. Compared to healthy controls, NLR values in SCZ are elevated [11] and are significantly higher in first-episode psychosis [13, 36] and during periods of relapse [37]. The precise mechanism of NLR elevation is unclear, however, and NLR alone cannot identify the precise source of inflammation. Here the influence of circadian regulation of endogenous cortisol and catecholamines (dopamine, noradrenaline, and epinephrine) play a role, as these hormones are known to increase neutrophil counts while decreasing lymphocyte counts [38]. In this case, elevated NLR in SCZ compared to healthy controls may provide an indication of sympathetic arousal in SCZ.

NLR is sensitive to psychotropic medications, with reports of reduced NLR values following antipsychotic treatment compared to medication-free patients [36, 37]. Nevertheless, many studies focus predominantly on the effect of antipsychotics on NLR, as reviewed by Sandberg [39] et al., despite treatment plans for SCZ more commonly including multiple psychiatric medications [11, 12]. We report that the association of SCZ with NLR was significant in all conditions, whether the patients were medicated or not or whether we adjusted for time of day or not. We report that the effect of medication on WBC was a general treatment effect, as medication regimes are personalized for optimal stabilization and recovery.

We adjusted for demographic and environmental covariates (sex, age, and smoking score) in the linear regression models, although we acknowledge that cellular proportions are also influenced by BMI, exercise, diet, and illness. We adjusted for a calculated smoking score rather than rely on self-reported smoking behavior. Despite the potential health risks of cannabis or illicit drug use, we were limited by self-reported use and did not adjust for their potential influence on WBC proportions. With health status in mind, we selected participants aged 18-48 years to avoid confounding due to potential health complications experienced later in life, including medications used in internal medicine, especially for patients.

Regarding the circadian cycling of WBC, our study was limited by available data for cases and controls within a 7-h time frame. In addition, archival whole blood DNA samples were used as we did not have access to actual cell counts. Ideally, a 24-h study with blood samples drawn at several intervals would be beneficial, and ultimately followed by a longitudinal study. Nevertheless, compared to flow cytometry, DNAm-based cell-type estimation derived from deconvolution methods is reproducible [40], and archival DNA and 5-methylcytosine are both stable [41].

Quality control of our methylation data was reinforced by the use of {Combat} for sensitivity and {SVA} for specificity [42]. These steps corrected sources of methylation variability in the data due to batch effects. The reference library selected for cell-type estimations by Salas [43] et al. uses a machine learning approach to optimize the accuracy of cell fraction estimates obtained from cell mixture deconvolution. This library is reported to have a consistently higher coefficient of determination (R2) and lower root mean square error (RMSE) compared to previous generations of deconvolution algorithms and methylation platforms. The reported estimation of methylation-derived NLR (mdNLR) is also consistent with previous findings from complete blood counts (CBC) where NLR was significantly higher in cancer [44] and positively associated with poor prognosis [45, 46].

A limitation of our models may be seen in the standard errors (SEs) (Supplementary Tables 5-8). Here the SEs were larger than the effect sizes for all cell types except for neutrophils, which represent the largest proportion of the six cell types and are more predominant early in the day [4]. The largest SEs were seen in the 12-h analysis suggesting a poor model fit, possibly due to fewer blood samples from cases in the afternoon. The SEs for NLR were largest for the medication-free samples, most likely due to the small sample size.

In our study, we sought to show that there was a general effect of polypharmacy on WBC counts compared to unmedicated samples. An expanded sample size of SCZ adhering solely to antipsychotics, however, would enhance our understanding of the specific effect of antipsychotics on cell counts, in contrast to detangling the potential anticholinergic burden of polypharmacy. Lastly, when evaluating the impact of polypharmacy on WBC proportions, it was not in the scope of this study to evaluate the half-lives of the different psychotropic drugs in the medicated samples nor to appraise the known influence of circadian rhythms on drug distribution in the blood [47]. Future longitudinal studies may enhance circadian research in psychiatric disorders, potentially contributing to the optimal time of day for medication efficacy.

## Conclusion

Our study reports that SCZ is significantly associated with changed proportions of neutrophils, CD4^+^T, CD8^+^T, and B-cells compared to healthy controls. This association remains significant after adjusting for time of day, although the effects are diminished compared to the unadjusted model. In addition, further investigation of medication effects is necessary to distinguish the biological effects attributable to SCZ more clearly from those attributable to treatment. Our results suggest that acknowledgment of time of day as an essential variable may enhance the accurate characterization of SCZ cases versus controls. Therefore, consideration of the temporal dimension in study design may contribute to this aim and promote much-needed reproducibility in biomedical research [48, 49].

## Supplementary information

Supplementary Tables and Figures

## References

[1] Hannon E, Dempster EL, Mansell G, Burrage J, Bass N, Bohlken MM (2021). DNA methylation meta-analysis reveals cellular alterations in psychosis and markers of treatment-resistant schizophrenia. eLife.

[2] Alvarez-Herrera S, Escamilla R, Medina-Contreras O, Saracco R, Flores Y, Hurtado-Alvarado G (2020). Immunoendocrine peripheral effects induced by atypical antipsychotics. Front Endocrinol.

[3] Zahorec R (2001). Ratio of neutrophil to lymphocyte counts—rapid and simple parameter of systemic inflammation and stress in critically ill. Bratisl Lek Listy.

[4] Pick R, He W, Chen C-S, Scheiermann C (2019). Time-of-day-dependent trafficking and function of leukocyte subsets. Trends Immunol.

[5] Beam CA, Wasserfall C, Woodwyk A, Akers M, Rauch H, Blok T (2020). Synchronization of the normal human peripheral immune system: a comprehensive circadian systems immunology analysis. Sci Rep.

[6] Delorme TC, Srivastava LK, Cermakian N (2020). Are circadian disturbances a core pathophysiological component of schizophrenia?. J Biol Rhythms.

[7] Walker WH, Walton JC, DeVries AC, Nelson RJ (2020). Circadian rhythm disruption and mental health. Transl Psychiatry.

[8] Ashton A, Jagannath A (2020). Disrupted sleep and circadian rhythms in schizophrenia and their interaction with dopamine signaling. Front Neurosci.

[9] Meyer N, Faulkner SM, McCutcheon RA, Pillinger T, Dijk D-J, MacCabe JH (2020). Sleep and circadian rhythm disturbance in remitted schizophrenia and bipolar disorder: a systematic review and meta-analysis. Schizophr Bull.

[10] Oh G, Koncevičius K, Ebrahimi S, Carlucci M, Groot DE, Nair A (2019). Circadian oscillations of cytosine modification in humans contribute to epigenetic variability, aging, and complex disease. Genome Biol.

[11] Karageorgiou V, Milas GP, Michopoulos I (2019). Neutrophil-to-lymphocyte ratio in schizophrenia: a systematic review and meta-analysis. Schizophr Res.

[12] Mazza MG, Lucchi S, Rossetti A, Clerici M (2020). Neutrophil-lymphocyte ratio, monocyte-lymphocyte ratio and platelet-lymphocyte ratio in non-affective psychosis: a meta-analysis and systematic review. World J Biol Psychiatry.

[13] Bioque M, Catarina Matias-Martins A, Llorca-Bofí V, Mezquida G, Cuesta MJ, Vieta E (2022). Neutrophil to lymphocyte ratio in patients with a first episode of psychosis: a two-year longitudinal follow-up study. Schizophr Bull.

[14] Vos CF, Birkenhäger TK, Nolen WA, van den Broek WW, Coenen MJH, ter Hark SE (2021). Association of the neutrophil to lymphocyte ratio and white blood cell count with response to pharmacotherapy in unipolar psychotic depression: an exploratory analysis. Brain Behav Immun - Health.

[15] First M, Spitzer R, Gibbon M, Williams J. Structured clinical interview for DSM-IV axis I disorders: patient edition (SCID-P), version 2. Biometrics Research (New York State Psychiatric Institute, 1995).

[16] Simonsen C, Sundet K, Vaskinn A, Birkenaes AB, Engh JA, Faerden A (2011). Neurocognitive dysfunction in bipolar and schizophrenia spectrum disorders depends on history of psychosis rather than diagnostic group. Schizophr Bull.

[17] Jónsdóttir H, Opjordsmoen S, Birkenaes AB, Simonsen C, Engh JA, Ringen PA (2013). Predictors of medication adherence in patients with schizophrenia and bipolar disorder: predictors of medication adherence. Acta Psychiatr Scand.

[18] Lin S-K (2020). Antipsychotic polypharmacy: a dirty little secret or a fashion?. Int J Neuropsychopharmacol.

[19] Stassen HH, Bachmann S, Bridler R, Cattapan K, Herzig D, Schneeberger A (2022). Detailing the effects of polypharmacy in psychiatry: longitudinal study of 320 patients hospitalized for depression or schizophrenia. Eur Arch Psychiatry Clin Neurosci.

[20] R Core Team. *R: A Language And Environment For Statistical Computing*. https://www.R-project.org/ (2020).

[21] Fortin JP, Triche TJ, Hansen KD (2017). Preprocessing, normalization and integration of the Illumina HumanMethylationEPIC array with minfi. Bioinformatics.

[22] Pidsley R, Zotenko E, Peters TJ, Lawrence MG, Risbridger GP, Molloy P (2016). Critical evaluation of the Illumina MethylationEPIC BeadChip microarray for whole-genome DNA methylation profiling. Genome Biol.

[23] Leek JT, Johnson WE, Parker HS, Jaffe AE, Storey JD (2012). The sva package for removing batch effects and other unwanted variation in high-throughput experiments. Bioinformatics.

[24] Tian Y, Morris TJ, Webster AP, Yang Z, Beck S, Feber A (2017). ChAMP: updated methylation analysis pipeline for Illumina BeadChips. Bioinformatics.

[25] Zhou W, Laird PW, Shen H (2017). Comprehensive characterization, annotation and innovative use of Infinium DNA methylation BeadChip probes. Nucleic Acids Res.

[26] The 1000 Genomes Project Consortium. (2015). A global reference for human genetic variation. Nature.

[27] Fortin J-P, Labbe A, Lemire M, Zanke BW, Hudson TJ, Fertig EJ (2014). Functional normalization of 450k methylation array data improves replication in large cancer studies. Genome Biol.

[28] Johnson WE, Li C, Rabinovic A (2007). Adjusting batch effects in microarray expression data using empirical Bayes methods. Biostatistics.

[29] Houseman EA, Accomando WP, Koestler DC, Christensen BC, Marsit CJ, Nelson HH (2012). DNA methylation arrays as surrogate measures of cell mixture distribution. BMC Bioinform.

[30] Salas L, Koestler D. *FlowSorted.Blood.EPIC*. https://github.com/immunomethylomics/FlowSorted.Blood.EPIC (2018).

[31] Vellame DS. *Deconvolution Specific Root Mean Squared Error (DSRMSE)*. https://github.com/ds420/DSRMSE (2020).

[32] Hannon E, Dempster E, Viana J, Burrage J, Smith AR, Macdonald R (2016). An integrated genetic-epigenetic analysis of schizophrenia: evidence for co-localization of genetic associations and differential DNA methylation. Genome Biol.

[33] Garcia-Rizo C, Casanovas M, Fernandez-Egea E, Oliveira C, Meseguer A, Cabrera B (2019). Blood cell count in antipsychotic-naive patients with non-affective psychosis. Early Inter Psychiatry.

[34] Jackson AJ, Miller BJ (2020). Meta‐analysis of total and differential white blood cell counts in schizophrenia. Acta Psychiatr Scand.

[35] Correll CU, Detraux J, De Lepeleire J, De Hert M (2015). Effects of antipsychotics, antidepressants and mood stabilizers on risk for physical diseases in people with schizophrenia, depression and bipolar disorder. World Psychiatry.

[36] Dawidowski B, Grelecki G, Biłgorajski A, Podwalski P, Misiak B, Samochowiec J (2021). Effect of antipsychotic treatment on neutrophil-to-lymphocyte ratio during hospitalization for acute psychosis in the course of schizophrenia—a cross-sectional retrospective study. JCM.

[37] Özdin S, Böke Ö (2019). Neutrophil/lymphocyte, platelet/lymphocyte and monocyte/lymphocyte ratios in different stages of schizophrenia. Psychiatry Res.

[38] Farkas JD (2020). The complete blood count to diagnose septic shock. J Thorac Dis.

[39] Sandberg AA, Steen VM, Torsvik A (2021). Is elevated neutrophil count and neutrophil-to-lymphocyte ratio a cause or consequence of schizophrenia?—a scoping review. Front Psychiatry.

[40] Titus AJ, Gallimore RM, Salas LA, Christensen BC (2017). Cell-type deconvolution from DNA methylation: a review of recent applications. Hum Mol Genet.

[41] Teschendorff AE, Zheng SC (2017). Cell-type deconvolution in epigenome-wide association studies: a review and recommendations. Epigenomics.

[42] Perrier F, Novoloaca A, Ambatipudi S, Baglietto L, Ghantous A, Perduca V (2018). Identifying and correcting epigenetics measurements for systematic sources of variation. Clin Epigenet.

[43] Salas LA, Koestler DC, Butler RA, Hansen HM, Wiencke JK, Kelsey KT (2018). An optimized library for reference-based deconvolution of whole-blood biospecimens assayed using the Illumina HumanMethylationEPIC BeadArray. Genome Biol.

[44] Aydın M, Bitkin A, Kadıhasanoğlu M, İrkılata L, Akgüneş E, Keleş M (2019). Correlation of neutrophil-lymphocyte ratio and risk scores in non-muscle invasive bladder cancer. Actas Urológicas Españolas (Engl Ed).

[45] Yuk HD, Jeong CW, Kwak C, Kim HH, Ku JH (2019). Elevated neutrophil to lymphocyte ratio predicts poor prognosis in non-muscle invasive bladder cancer patients: initial intravesical bacillus Calmette-Guerin treatment after transurethral resection of bladder tumor setting. Front Oncol.

[46] Getzler I, Bahouth Z, Nativ O, Rubinstein J, Halachmi S (2018). Preoperative neutrophil to lymphocyte ratio improves recurrence prediction of non-muscle invasive bladder cancer. BMC Urol.

[47] Dong D, Yang D, Lin L, Wang S, Wu B (2020). Circadian rhythm in pharmacokinetics and its relevance to chronotherapy. Biochem Pharmacol.

[48] Oh ES, Petronis A (2021). Origins of human disease: the chrono-epigenetic perspective. Nat Rev Genet.

[49] Nelson RJ, Bumgarner JR, Liu JA, Love JA, Meléndez-Fernández OH, Becker-Krail DD (2022). Time of day as a critical variable in biology. BMC Biol.

