# Supplementary material for: Differences in white blood cell proportions between schizophrenia cases and controls are influenced by medication and variations in time of day
Source: Transl Psychiatry. 2023 Jun 17;13:211. doi: 10.1038/s41398-023-02507-1 (PMC10276856; doi:10.1038/s41398-023-02507-1)

**Supplementary Tables and Figures**

**Differences in white blood cell proportions between schizophrenia cases and controls are influenced by medication and variations in time of day**

Jonelle Villar, Anne-Kristin Stavrum, Letícia Spíndola, Anja Torsvik, Thomas Bjella, Niels Eiel Steen, Srdjan Djurovic, Ole A. Andreassen, Vidar M. Steen, and Stéphanie Le Hellard

Supplementary Table 1 page 2

Supplementary Table 2 page 3

Supplementary Table 3 page 4

Supplementary Table 4 page 5

Supplementary Table 5 page 6

Supplementary Table 6 page 7

Supplementary Table 7 page 8

Supplementary Table 8 page 9

Supplementary Figure 1 page 10

Supplementary Figure 2 page 11

Supplementary Figure 3 page 11

Supplementary Table 1. Cases vs Controls: 12-hour day (07:00-19:00). “Hrs_from_07” represents 07:00 as a baseline and the number of hours from baseline when the blood draw was taken.


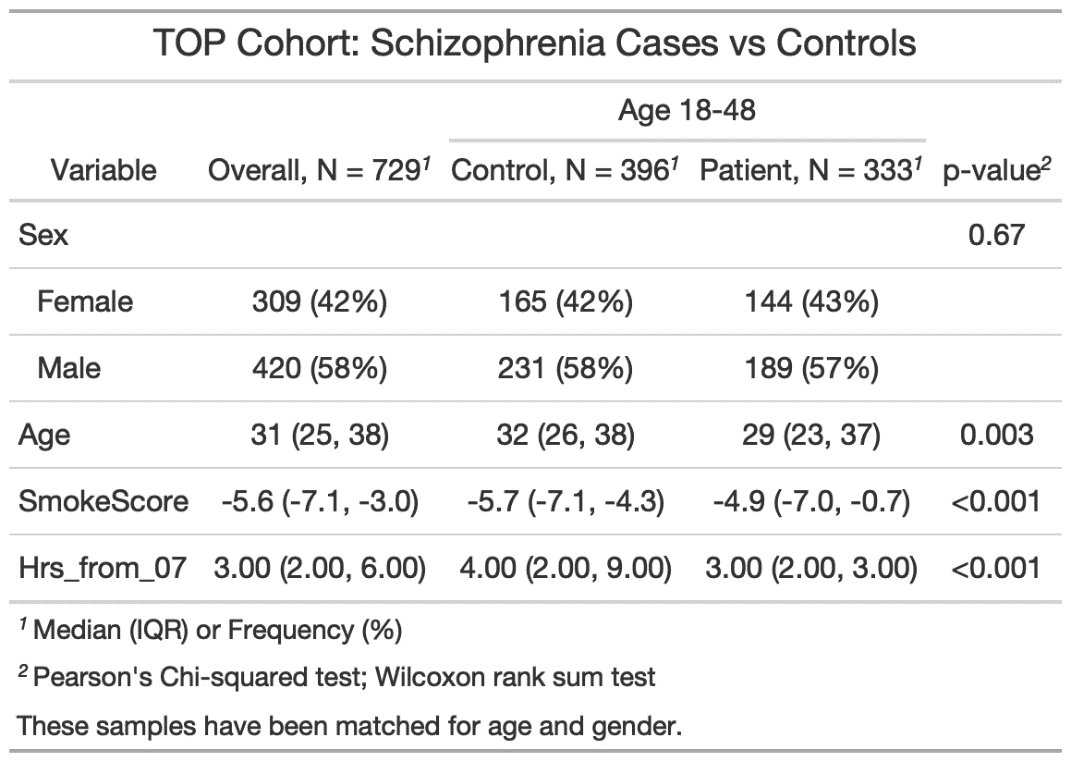


Supplementary Table 2. 7-hour day (07:00-14:00). The variable “hourDiff” represents 07:00 as a baseline and the number of hours from baseline when the blood draw was taken.


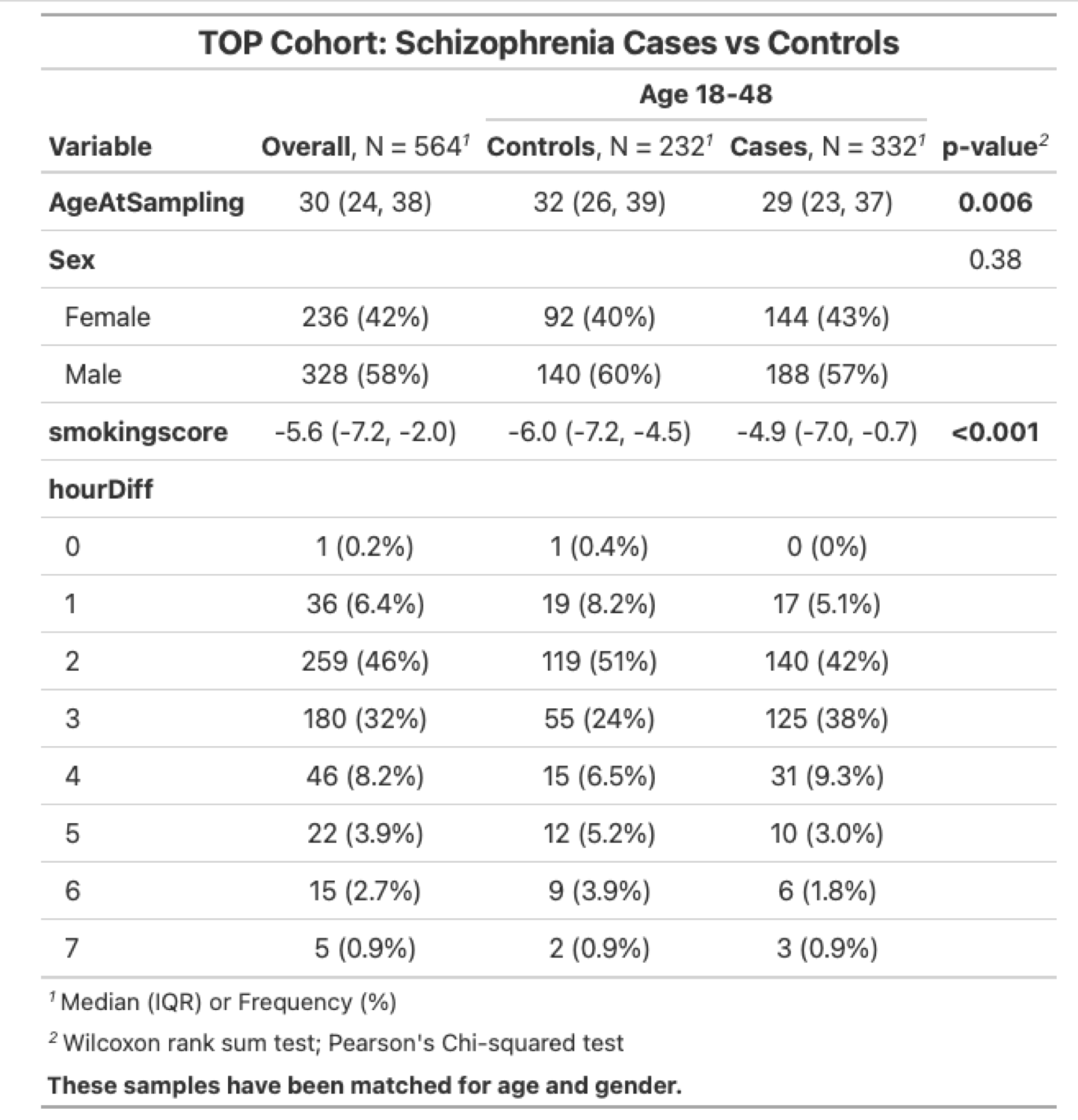


Supplementary Table 3. 12-hour day– Medication-free cases vs. controls. “Hrs_from_07” represents 07:00 as a baseline and the number of hours from baseline when the blood draw was taken.


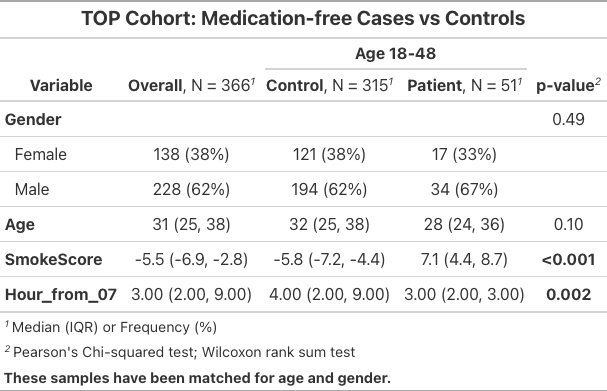


Supplementary Table 4. – 7-hour day for Medication-free cases vs. controls. “Hrs_from_07” represents 07:00 as a baseline and the number of hours from baseline when the blood draw was taken.


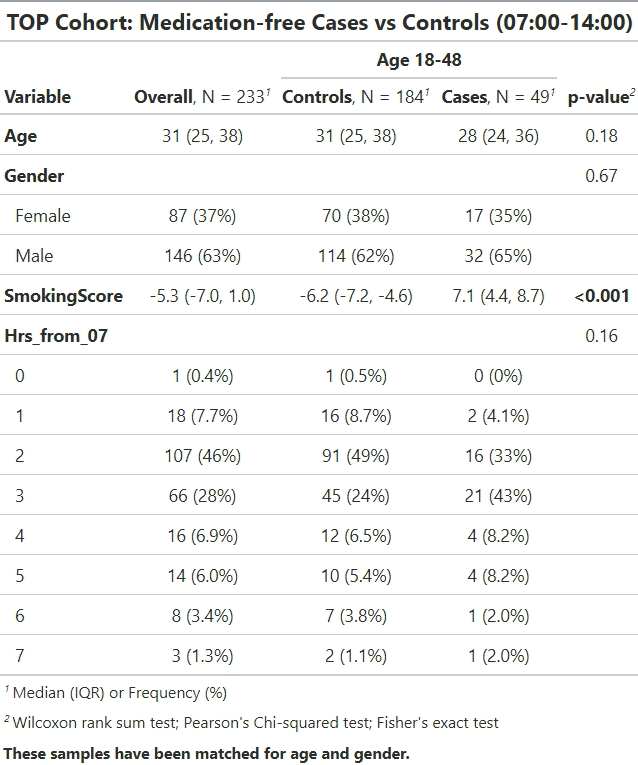


Supplementary Table 5. – Influence of SCZ status and time of blood draw on cell proportions in a 12-hour day (07:00-19:00).


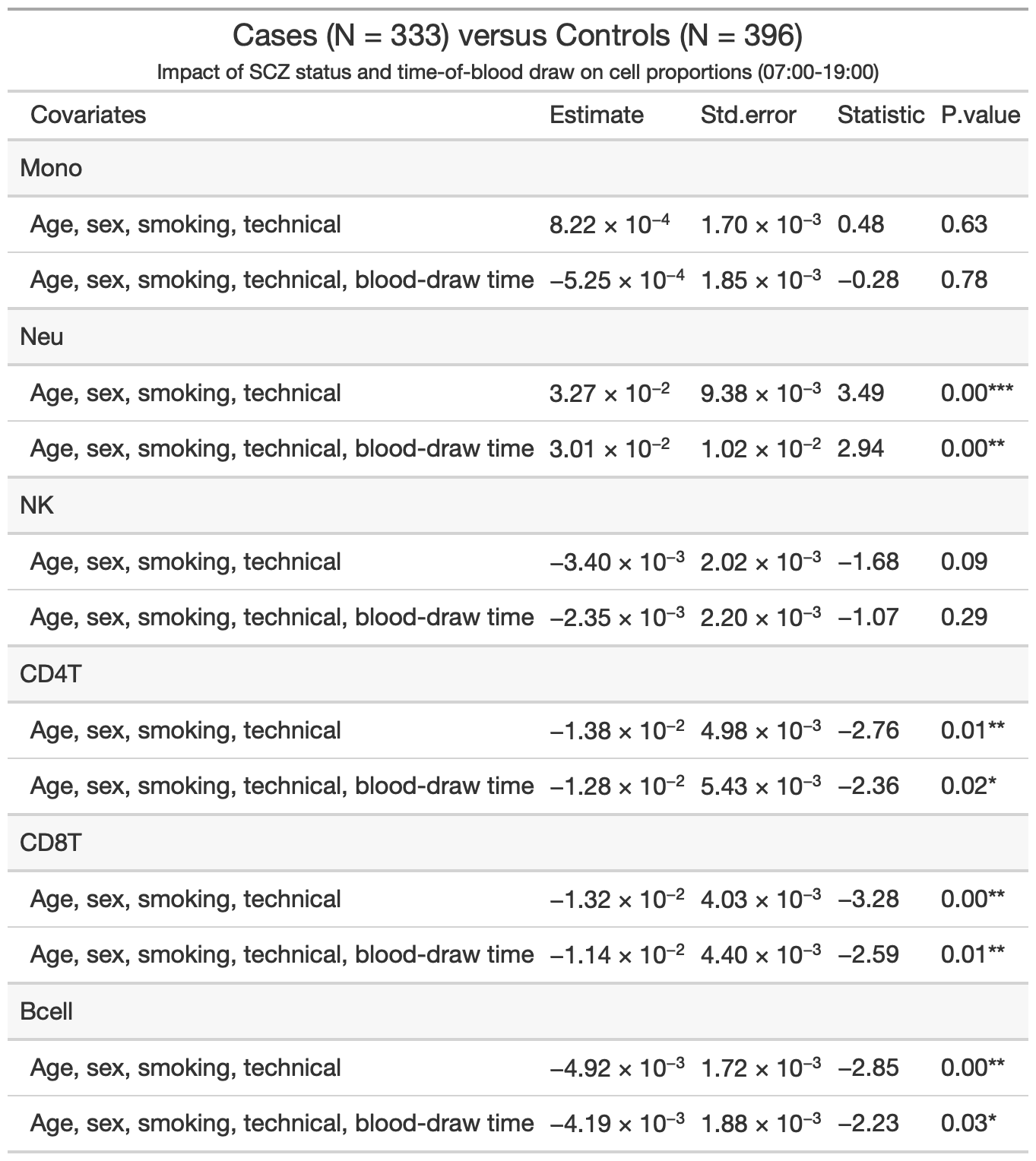


Supplementary Table 6. Influence of SCZ status and time of blood draw on cell proportions in a 7-hour day (07:00-14:00).


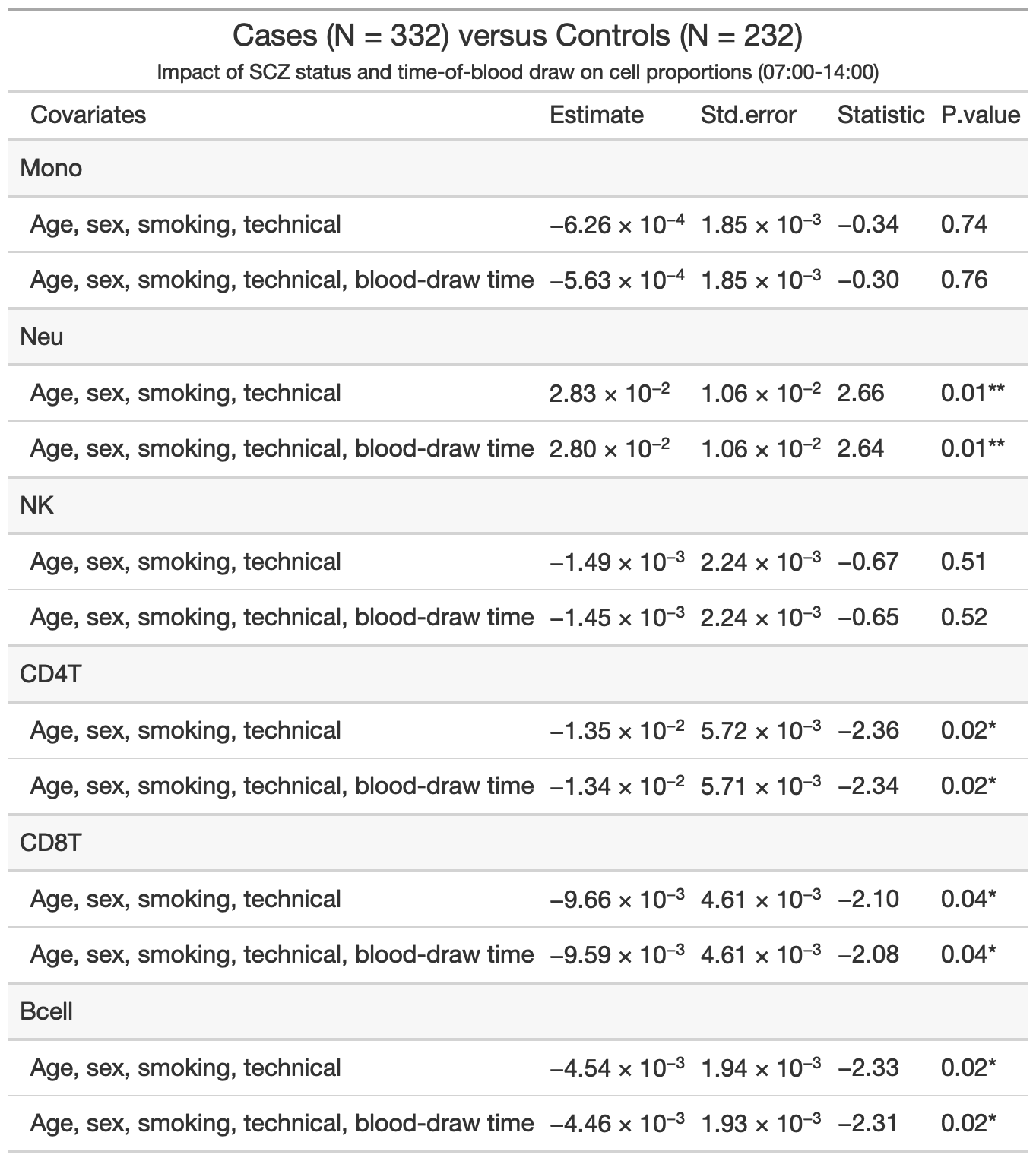


Supplementary Table 7. Influence of Medication-free SCZ status and time of blood draw on cell proportions in a 12-hour day (07:00-19:00).


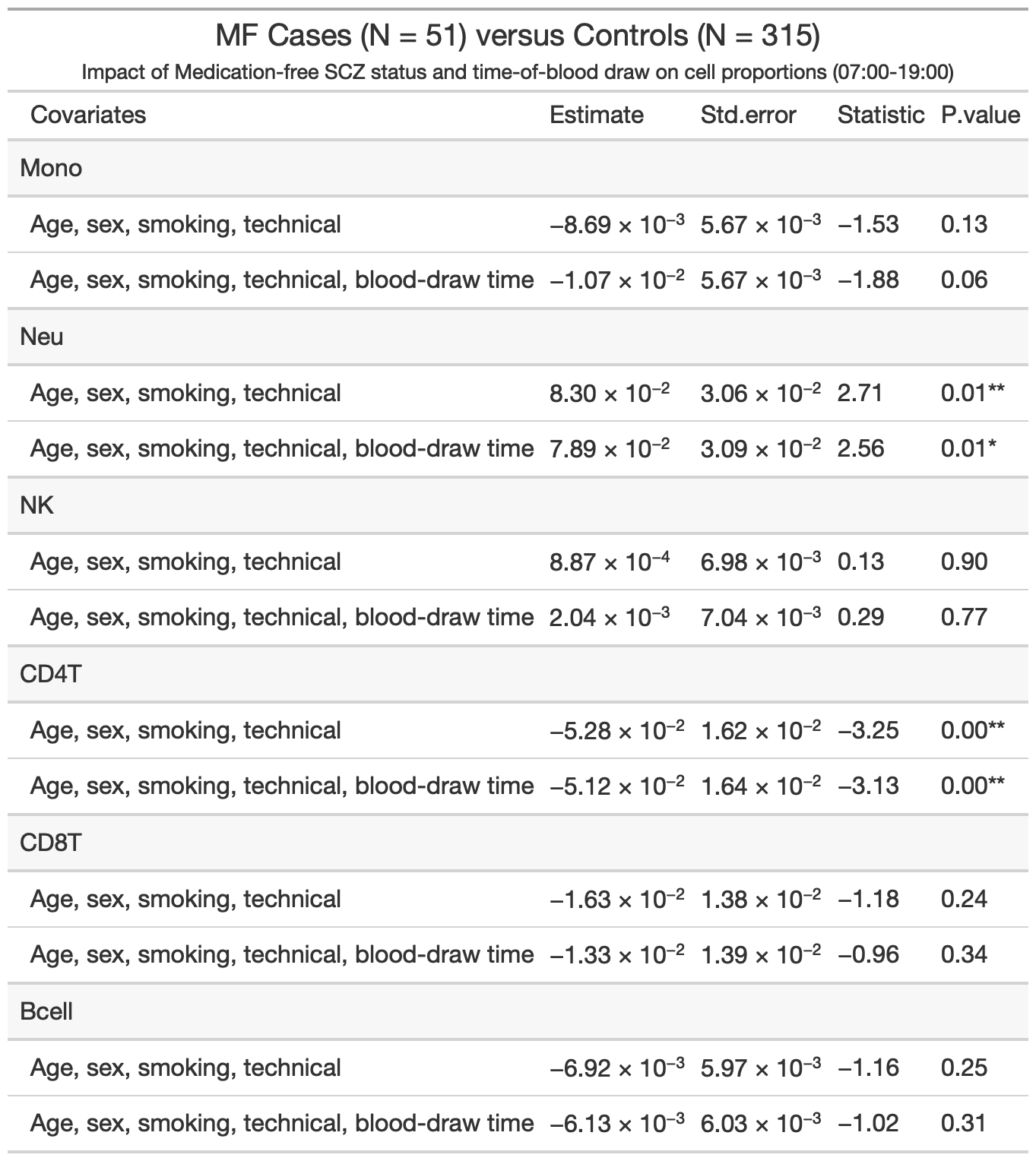


Supplementary Table 8. Influence of Medication-free SCZ status and time of blood draw on cell proportions in a 7-hour day (07:00-14:00).


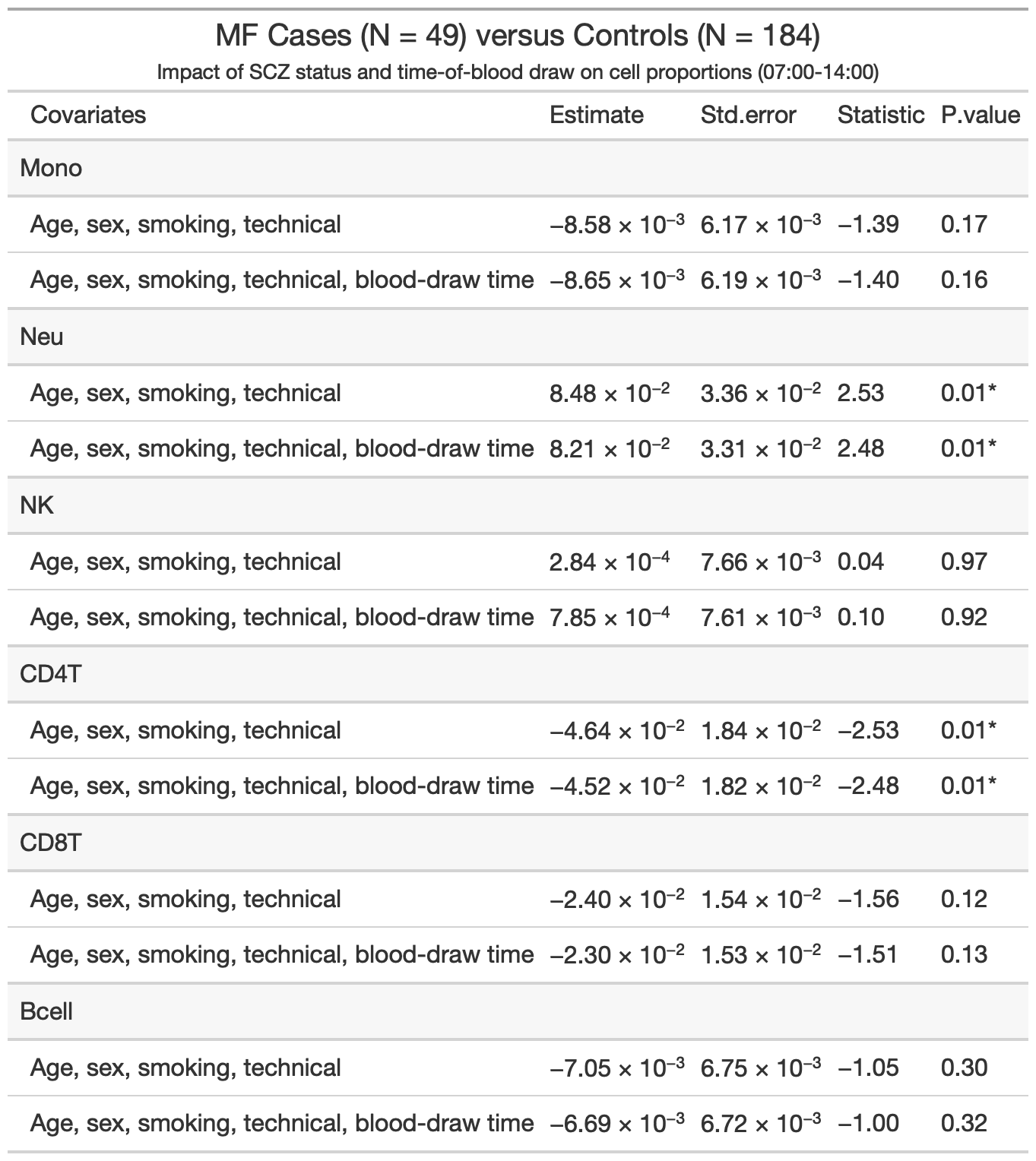


Supplementary Figure 1. Neutrophil-to-lymphocyte ratio. Two samples were removed as outliers with NLR > 4.00. Cases vs. Controls


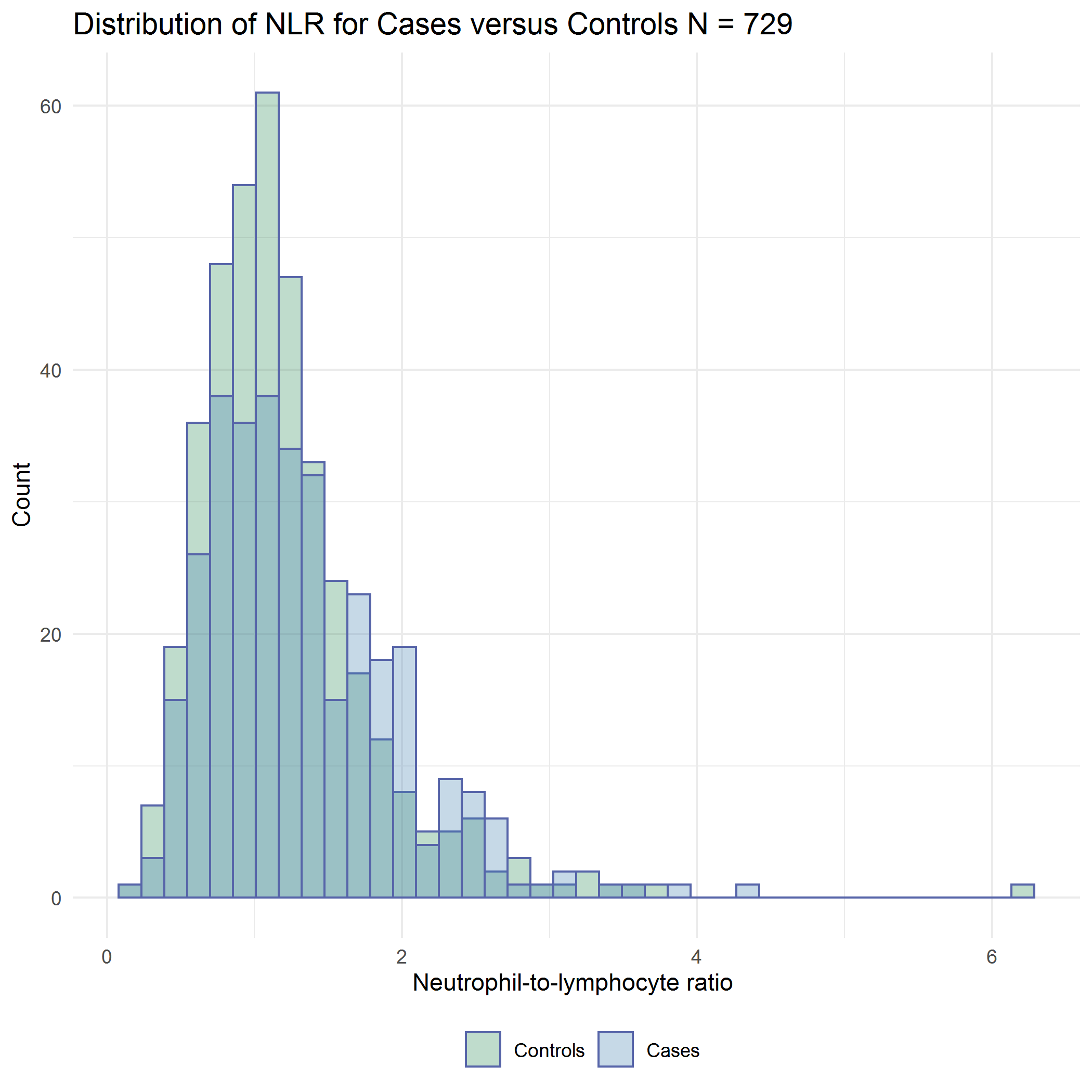


Supplementary Figure 2. Distribution of time of blood draw: Cases vs. Controls 7-hour day (07:00-14:00)


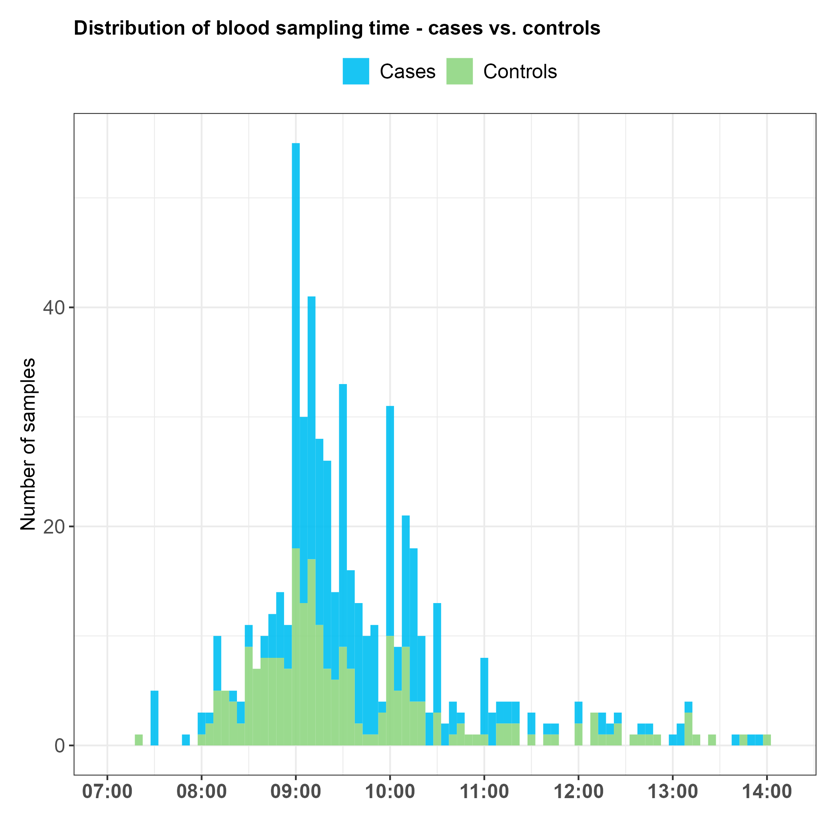


Supplementary Figure 3. Distribution of time of blood draw: Medication-free vs. Controls 7-hour day (07:00-14:00)


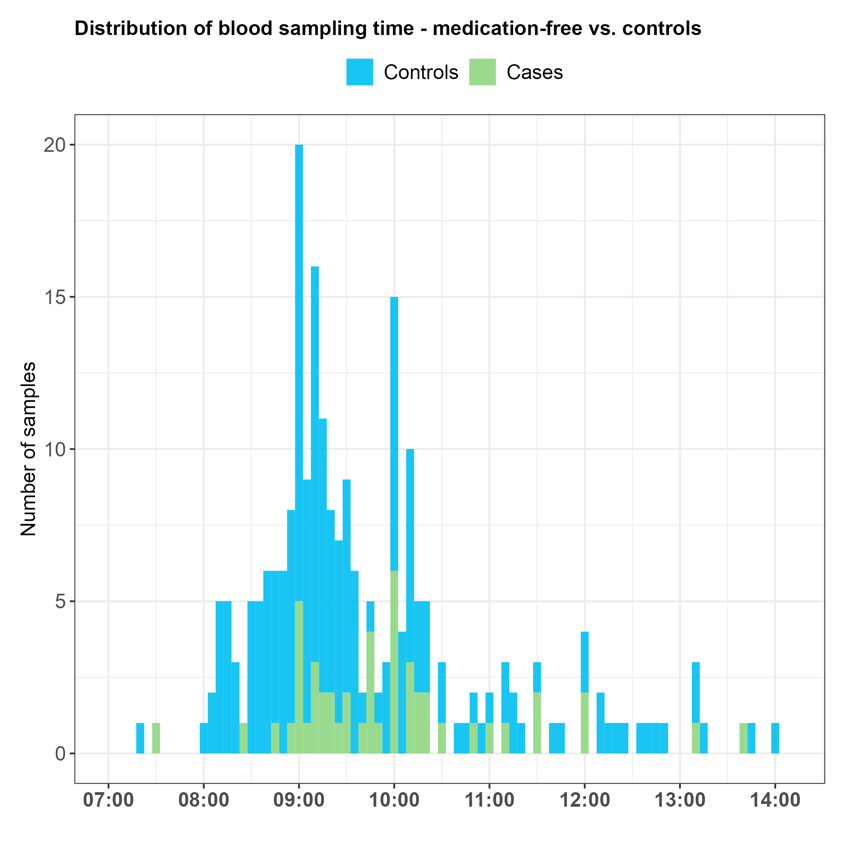

Supplement: Supplementary file 1 — Supplementary Tables and Figures [file 41398_2023_2507_MOESM1_ESM.docx]
